# Two nomograms constructed for predicting the efficacy and prognosis of advanced non‑small cell lung cancer patients treated with anti‑PD‑1 inhibitors based on the absolute counts of lymphocyte subsets

**DOI:** 10.1007/s00262-024-03738-x

**Published:** 2024-06-04

**Authors:** Aqing Liu, Guan Zhang, Yanjie Yang, Ying Xia, Wentao Li, Yunhe Liu, Qian Cui, Dong Wang, Jianchun Yu

**Affiliations:** 1 Department of Oncology, National Clinical Research Center for Chinese Medicine Acupuncture and Moxibustion, First Teaching Hospital of Tianjin University of Traditional Chinese Medicine, Tianjin, China; 2 Department of Oncology, First Affiliated Hospital of Xi’an Jiaotong University, Xi’an, China; 3 Graduate School, Tianjin University of Traditional Chinese Medicine, Tianjin, China

**Keywords:** Lymphocyte subsets, Absolute count, Non-small cell lung cancer, Anti-PD-1inhibitor, Nomogram

## Abstract

**Background:**

Patients treated with immune checkpoint inhibitors (ICIs) are at risk of considerable adverse events, and the ongoing struggle is to accurately identify the subset of patients who will benefit. Lymphocyte subsets play a pivotal role in the antitumor response, this study attempted to combine the absolute counts of lymphocyte subsets (ACLS) with the clinicopathological parameters to construct nomograms to accurately predict the prognosis of advanced non-small cell lung cancer (aNSCLC) patients treated with anti-PD-1 inhibitors.

**Methods:**

This retrospective study included a training cohort (n = 200) and validation cohort (n = 100) with aNSCLC patients treated with anti-PD-1 inhibitors. Logistic and Cox regression were conducted to identify factors associated with efficacy and progression-free survival (PFS) respectively. Nomograms were built based on independent influencing factors, and assessed by the concordance index (C-index), calibration curve and receiver operating characteristic (ROC) curve.

**Result:**

In training cohort, lower baseline absolute counts of CD3^+^ (*P *< 0.001) and CD4^+^ (*P* < 0.001) were associated with for poorer efficacy. Hepatic metastases (*P* = 0.019) and lower baseline absolute counts of CD3^+^ (*P* < 0.001), CD4^+^ (*P* < 0.001), CD8^+^ (*P* < 0.001), and B cells (*P* = 0.042) were associated with shorter PFS. Two nomograms to predict efficacy at 6-week after treatment and PFS at 4-, 8- and 12-months were constructed, and validated in validation cohort. The area under the ROC curve (AUC-ROC) of nomogram to predict response was 0.908 in training cohort and 0.984 in validation cohort. The C-index of nomogram to predict PFS was 0.825 in training cohort and 0.832 in validation cohort. AUC-ROC illustrated the nomograms had excellent discriminative ability. Calibration curves showed a superior consistence between the nomogram predicted probability and actual observation.

**Conclusion:**

We constructed two nomogram based on ACLS to help clinicians screen of patients with possible benefit and make individualized treatment decisions by accurately predicting efficacy and PFS for advanced NSCLC patient treated with anti-PD-1 inhibitors.

## Introduction

Lung cancer represents the leading cause of cancer-mortality worldwide [1]. More than 85% of cases are classified as non-small-cell lung cancer (NSCLC), with predicted 5-year survival of 16% [2]. Therefore, the prevention and treatment of NSCLC has become an urgent global health issue. However, most NSCLC patients are diagnosed at an advanced stage and miss the best opportunity for surgical treatment [3]. The main treatments for patients with advanced NSCLC (aNSCLC) are radiotherapy, chemotherapy and targeted therapy, but most patients cannot tolerate the side effects of radiotherapy and chemotherapy [4], and targeted therapy is highly susceptible to drug resistance [5], so the beneficiary patients are limited.

In recent years, immune checkpoint inhibitors (ICIs), a new type of anti-tumor immunotherapy, have made breakthroughs in several areas of cancer treatment, including lung cancer [3]^.^ ICIs targeting programmed death-1 (PD-1) have revolutionized the prognosis of advanced NSCLC patients [6] and many other malignancies [7]. Anti-PD-1 antibodies are mainly represented by nivolumab and pembrolizumab, which could significantly prolong the overall survival (OS) and progression-free survival (PFS) in NSCLC patients compared with the second-line treatment of chemotherapy [8–10]. In 2020, the Chinese Society of Clinical Oncology recommended camrelizumab as the first-line treatment for EGFR/ALK mutation-negative advanced non-squamous NSCLC [11]. Although ICIs has dramatically changed the treatment landscape for cancer patients, not all patients can benefit from it. Studies have shown that only 15% of NSCLC patients benefit from ICIs monotherapy, and patients receiving immunotherapy are at risk of considerable immune-related adverse events (iRAEs) or even hyperprogression disease [12, 13]. Therefore, it is urgent to accurately identify the subset of patients who benefit.

At present, more and more biomarkers have been used to predict the efficacy of immunotherapy, including programmed death protein ligand-1 (PD-L1), blood-based tumor mutation burden (bTMB), microsatellite instability (MSI), DNA damage repair (DDR), tumor-infiltrating lymphocytes (TILs), etc. [14, 15]^.^ However, these biomarkers are hampered by the difficulties in obtaining the organization and technical challenges, and it is difficult to separate beneficial patients from non-beneficial patients. Although immunohistochemical detection of PD-L1 expression is currently the most widely used method to predict the efficacy of ICIs [16], this method has the disadvantages of subjectivity and internal tumor heterogeneity. The Keynote-024 study found that patients with PD-L1 expression ≥ 50% benefited from pembrolizumab [17], while the CheckMate 026 study found that the efficacy of patients with PD-L1 expression ≥ 5% was not associated with nivolumab [18]. The prognosis is not determined by an isolated factor only. Studies found that other independent prognostic factors such as gender, age, histology and treatment-related factors can significantly influence the individualized survival prediction [19, 20]. Therefore, it is important to explore more effective methods to predict the efficacy of immunotherapy.

The lymphocyte subsets, are a very important heterogeneous population of immune cells which mainly including T, B and natural killer cells (NK), play key roles in the immune recognition, response and clearance of tumors and pathogens [21–23]. Studies have shown that a reduction in baseline peripheral blood absolute lymphocyte count was closely associated with the efficacy of immunotherapy and poor prognosis in cancer patients [24, 25]. Our previous study revealed that baseline peripheral blood absolute counts of lymphocyte subsets (ACLS) were significantly lower in NSCLC patients compared to normal subjects, and the lower the ACLS, the worse the prognosis [26]. However, whether ACLS can accurately predict efficacy and progression-free survival (PFS) for aNSCLC patients receiving anti-PD-1 inhibitors remains unclear.

Nomogram is a reliable and convenient prognostic tool to predict prognosis of cancer patients. By quantifying and integrating different prognostic factors, it can provide accurate individualized prognostic prediction, and has a better predictive ability than the traditional TNM staging [27].

Therefore, the study aimed to construct nomograms to accurately predict the efficacy and PFS receiving anti-PD-1 inhibitor therapy by investigating the predictive and prognostic value of ACLS combined with clinicopathological parameters associated with anti-PD-1 inhibitors and PFS.

## Patients and methods

### Patients selection

A single-center retrospective study of total 300 aNSCLC patients received anti-PD-1 inhibitors screening from 2498 NSCLC patients between January 2018 and January 2021 from Department of Oncology, First Teaching Hospital of Tianjin University of Traditional Chinese Medicine was conducted. The inclusion criteria were as follows: (1) patients aged 18–80 years; (2) with complete electronic medical record including ACLS; (3) pathologically diagnosed with NSCLC; (4) with clinical stage IIIB/IV; (5) with a baseline Eastern Cooperative Oncology Group (ECOG) performance-status score of 0 or 1 and expected survival ≥ 6 months; (6) at least two cycles of anti-PD-1 inhibitors monotherapy, and (6) without other malignant tumors. Furthermore, patients were excluded if they met the following exclusion criteria: (1) undetermined diagnosis of NSCLC; (2) combined with other malignant tumors; (3) with *EGFR/ALK* alterations; (4) had missing clinical data; and (5) underlying conditions such as acute infection, hematological disorders, autoimmune diseases, pregnancy or lactation.

**Fig. 1:**
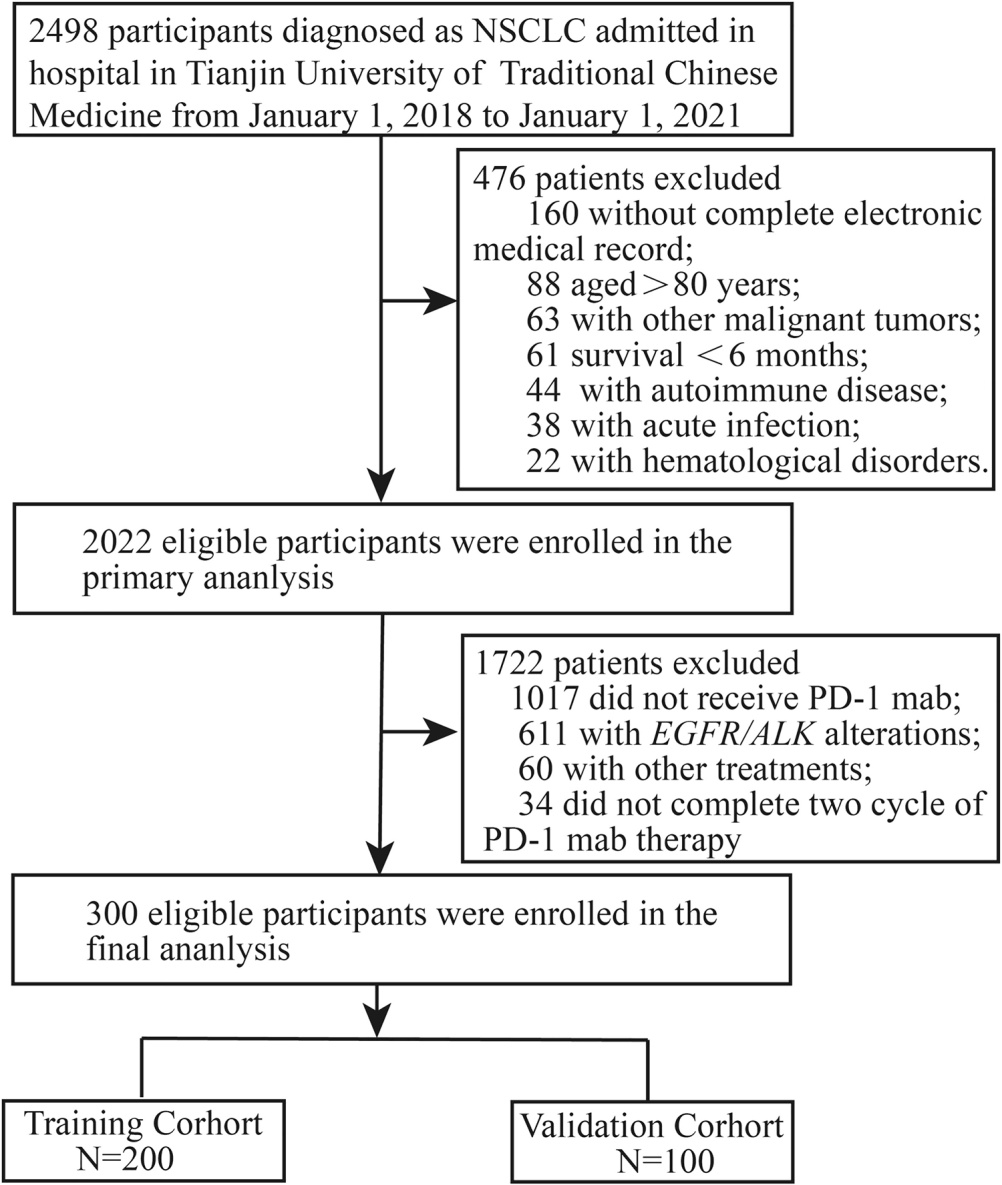
Patient screening flow chart

A total of 300 patients who met the inclusion criteria were randomly assigned to the training and validation cohort at a 2:1 ratio. The patients selection process and study design were shown in Fig. 1. The study was conducted in accordance with the Declaration of Helsinki and approved by the Clinical Research Ethics Committee of First Teaching Hospital, Tianjin University of Traditional Chinese Medicine (TYLL2017[K]002, 25 December 2017, Tianjin, China) and registered at Chinese Clinic Trial Registry (ChiCTR-IOR-17014139).

### Data collection

Clinicopathological data, including demographic information (age, gender), smoking history, tumor size, histological type, differentiation, lymphatic metastasis, distant metastases (bone, brain, and liver metastases), clinical stage (stage III, IV), treatments, progression-free survival (PFS), and immunological parameters (baseline absolute counts and percentage of CD3^+^, CD4^+^, CD8^+^, B, and NK cells), were collected.

### Treatment

Camrelizumab and sintilimab were administered intravenously at a dose of 200 mg every 3 weeks, respectively. Pembrolizumab was administered intravenously at a dose of 2 mg/kg every 3 weeks.

### Study endpoints

The study endpoints were objective response rate (ORR) and PFS. The efficacy of anti-PD-1 inhibitors was evaluated by computed tomography or magnetic resonance imaging according to the Response Evaluation Criteria in Solid Tumors (RECIST version 1.1) [28]. The ORR was defined as the proportion of patients with complete response (CR) and partial response (PR) to all evaluable patients after treatment. PFS was defined as the time from the start of the treatment until the date of disease progression, death or last follow-up (censored data).

### The main reagents and instruments

Lymphocyte subsets were assayed by using a lyse/no-wash procedure based on a single-platform technique of ten-color flow cytometry (BD FACS Canto II: U6573380-00541, USA). The reagents were BD Multitest IMK kit (Catalog NO: 662,965) containing (BD Multitest CD3FITC/CD8PE/CD45PerCP/CD4APC and BD Multitest CD3 FITC/CD16^+^CD56^+^PE/ CD45PerCP/CD19APC), and BD Multitest IMK kit lysing solution (Catalog NO: 91–1087). The EDTA blood collecting tubes and trucount tubes (Catalog NO: 340,334) were also purchased from BD Biosciences, USA.

### Sample collection

Two milliliters of fresh whole blood drawn from the NSCLC patients were stored in the EDTA-anticoagulant blood collecting tubes.

### Cellular staining and analyzing

Whole blood of the 300 participants was collected and assayed by flow cytometry following the BD operating instruction. In brief, for each sample, two trucount tubes were labeled with letters A and B to distinguish them from each other. 20 μL of BD Multitest CD3^+^/CD4^+^/CD8^+^/CD45^+^ and CD3^−^/CD16^+^CD56^+^/CD45^+^/CD19^+^ reagents were added to the bottom of tubes A and B, respectively. Then, 50 μL of well-mixed whole blood was added to the bottom of every tube. Finally, 450 μL of lysing solution was added into every tube, mixed, and incubated for 15 min in the dark at room temperature for analysis.

### Statistical analysis

The X-tile (Yale University, version 3.6.1) software was used to determine the optimal cutoff values of absolute count of CD3^+^, CD4^+^, CD8^+^, B and NK cells based on the patient's PFS. Continuous variables were expressed as mean ± SD or median, and categorized variables were described by the numbers and percentages. The chi-square test was used for the comparison of categorical variables between the two groups. Independent t-test was used for the comparison of continuous numerical variables between the two groups. In the training cohort, the associations of variables with efficacy and PFS were analyzed. Univariate and multivariate logistic regression analysis were performed to assess the relationship between clinicopathological parameters and efficacy. Univariate cox proportional hazard regression analysis was performed to explore significant factors associated with PFS of aNSCLC patients. Variables with a *P* < 0.05 in the univariate analysis were entered into multivariate cox proportional hazard regression analysis to determine which variables were the independent factors that were associated with PFS. Survival analysis was performed using the Kaplan–Meier method with log-rank test to evaluate the differences in PFS between groups.

### Development, validation, and evaluation of the nomograms

Variables with a *P* < 0.05 in the multivariate logistic regression analysis were eventually included in the construction of the nomogram for predicting efficacy of anti-PD-1 inhibitors. Moreover, variables with a *P* < 0.05 in the multivariate Cox regression analysis were ultimately included in the development of the nomogram for predicting PFS. The two nomograms were built to predict the efficacy probability at 6 weeks after treatment and the probability of PFS at 4-, 8- and 12- months after anti-PD-1 inhibitors treatment in advanced NSCLC patients based on the predictors identified in the training cohort, respectively. Then, the nomograms were validated internally by using bootstrap method with 1000 resamples and externally in the validation cohorts. The performance of the nomogram was evaluated using a concordance index (C-index), the area under the receiver operating characteristic curve (AUC-ROC) and calibration curve [29]. The C-index and AUC-ROC were used to assess the discriminatory ability of the nomogram. The calibration curve was utilized to evaluate the level of agreement between the predicted survival probability and the actual observed survival probability.

Statistical analyses and graphing were carried out using the packages of “survival”, “survminer”,“rms”, “lrm”, and “survivalROC” in R version 4.1.1 (http://www.r-project.org/) for logistic regression and Cox regression, forest plot, survival analysis, nomogram generation, ROC curve analysis, C-index assessment, and calibration curve generation. The rest of the statistical analyses were performed using SPSS version 25.0 (IBM Corporation, Armonk, NY) and graphing using Graphpad Prism 9.0 (San Diego, USA). All statistical tests were two-tailed, and *P* < 0.05 was considered statistically significant.

## Results

### Patients’ characteristics

A total of 300 aNSCLC patients were included in accordance with the inclusion criteria, including 200 patients in the training cohort and 100 patients in the validation cohort. The median PFS was 4 months (range 1–18 months). The median age was 66 years (range 31–80 years). 219 patients (73.0%) were male. There were 236 patients (78.7%) with stage IV disease, and 220 patients (73.3%) had a prior history of smoking. 148 patients (49.3%) received camrelizumab, 102 patients (34.0%) treated by pembrolizumab, and 50 patients (16.7%) exposed to sintilimab. The ORR was 19.3%. In terms of histology, 187 patients (62.3%) had adenocarcinoma, 84 patients (28.0%) had squamous carcinoma, and 29 patients (9.7%) had other types (large cell carcinoma and adenosquamous carcinoma). The baseline characteristics are described in Table 1. All baseline clinicopathologic characteristics and immunology parameters were comparable in the training and validation cohorts.

**Table 1:** Baseline characteristics of patients

Characteristics	All patients		Training cohort		Validation cohort	
	(N = 300)		(N = 200)		(N = 100)	
	Number	%	Number	%	Number	%
Age(years)						
Mean	65.48		65.35		65.73	
Median	66		65.5		67	
Range	31–80		32–80		31–79	
Gender						
Male	219	73.0	143	71.5	76	76.0
Female	81	27.0	57	28.5	24	24.0
Smoking history						
No	80	26.7	55	25.5	25	25.0
Yes	220	73.3	145	74.3	75	75.0
Tumor size						
< 2cm	44	14.7	29	14.5	15	15
≥ 2cm	256	85.3	171	85.5	85	85
Histology						
Adenocarcinoma	187	62.3	123	61.5	64	64.0
Squamous carcinoma	84	28.0	59	29.5	25	25.0
Others	29	9.7	18	9.0	11	11.0
Differentiation						
Median/high	63	21.0	40	20.0	23	23.0
Low	237	79.0	160	80.0	77	77.0
Clinical stages						
III	64	21.3	43	21.5	21	21.0
IV	236	78.7	157	78.5	79	79.0
Lymphatic metastasis						
No	62	20.7	45	22.5	17	17.0
Yes	238	79.3	155	77.5	83	83.0
Brain metastases						
No	220	73.3	150	75.0	70	70.0
Yes	80	26.7	50	25.0	30	30.0
Bone metastases						
No	170	56.7	116	58.0	54	54.0
Yes	130	43.3	84	42.0	46	46.0
Hepatic metastases						
No	221	73.7	147	73.5	74	74.0
Yes	79	26.3	53	26.5	26	26.0
PD-L1 expression						
Negative	152	50.7	102	51.0	50	50.0
Positive	138	46.0	91	45.5	47	47.0
Unknown	10	3.3	7	3.5	3	3.0
Treatment						
Camrelizumab	148	49.3	96	48.0	46	46.0
Pembrolizumab	102	34.0	64	32.0	35	35.0
Sintilimab	50	16.7	40	20.0	19	19.0
Curative effect						
CR	0	0.0	0	0.0	0	0.0
PR	58	19.3	43	21.5	15	15.0
SD	92	30.7	68	34.0	24	24.0
PD	150	50.0	89	44.5	61	61.0
Immunological parameters						
CD3^+^ %	69.3		69.0		69.9	
CD4^+^ %	39.9		40.3		39.1	
CD8^+^ %	23.5		24.1		22.3	
B %	11.0		10.6		11.9	
NK %	13.5		13.1		14.4	
CD3^+^AC (cells/μL)	737.5		746.6		719.4	
CD4^+^AC (cells/μL)	398.0		402.3		389.5	
CD8^+^AC (cells/μL)	236.6		242.8		224.1	
B AC (cells/μL)	119.0		116.4		124.1	
NKAC (cells/μL)	145.2		146.3		143.0	
PFS (months)						
Mean	6.12		6.57		5.51	
Median	4.00		5.00		4.00	
Range	1–18		1–18		1–18	

### The correlation between ACLS and curative effect

**Fig. 2:**
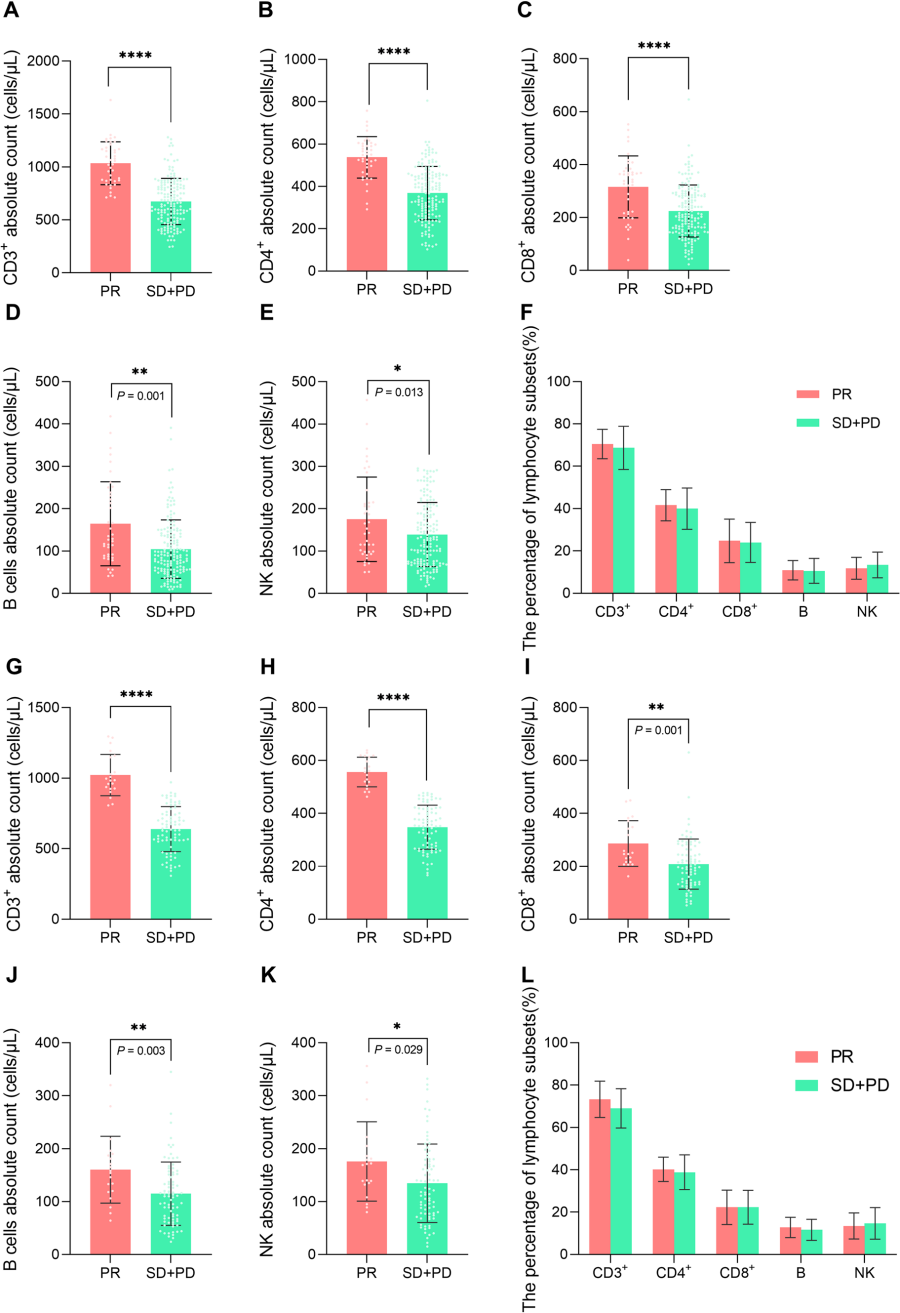
The comparison of percentage and absolute counts of lymphocyte subsets between response (PR) and nonresponse (SD + PD) groups in training and validation cohorts. The comparison of absolute count of CD3^+^ (**A**), CD4^+^ (**B**), CD8^+^ (**C**), B (**D**), NK (**E**) cells and the percentage of lymphocyte subsets (**F**) between response and nonresponse group in the training cohort. The comparison of absolute count of CD3^+^ (**G**), CD4^+^ (**H**), CD8^+^ (**I**), B (**J**), NK (**K**) cells and the percentage of lymphocyte subsets (**L**) between response and nonresponse groups in the validation cohort. *P < 0.05, **P < 0.01, ***P < 0.001, ****P < 0.0001

To clarify the correlation between the ACLS, percentage and the therapeutic response of anti-PD-1 antibody, the difference of absolute counts (AC) and percentage of CD3^+^, CD4^+^, CD8^+^, B and NK cells was compared between the response and nonresponse groups in training and validation cohort. The results showed that the absolute counts of CD3^+^, CD4^+^, CD8^+^, B, and NK cells in the response group were significantly higher than those in the nonresponse group in both the training and validation cohorts (*P* < 0.05) (Fig. 2A–D and G–K), while the percentage of the lymphocyte subsets above was not significantly different in both cohorts (*P* > 0.05) (Fig. 2F, L). These suggested that it was the ACLS but not the percentage of the lymphocyte subsets that positively correlated with the efficacy of anti-PD-1 inhibitors. The higher the ACLS, the better the efficacy, vice versa.

### The correlation between the ACLS and PFS

**Fig. 3:**
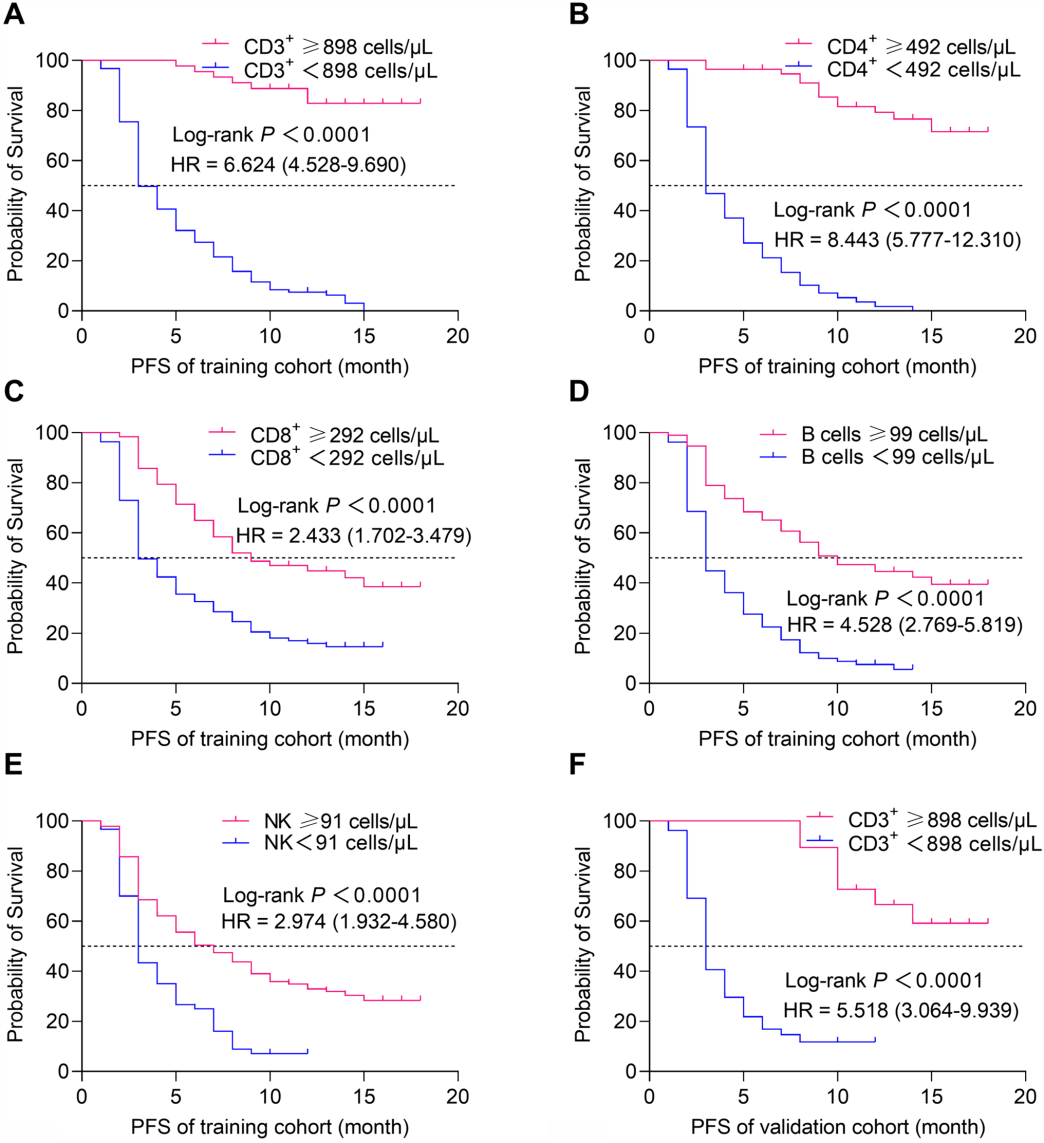

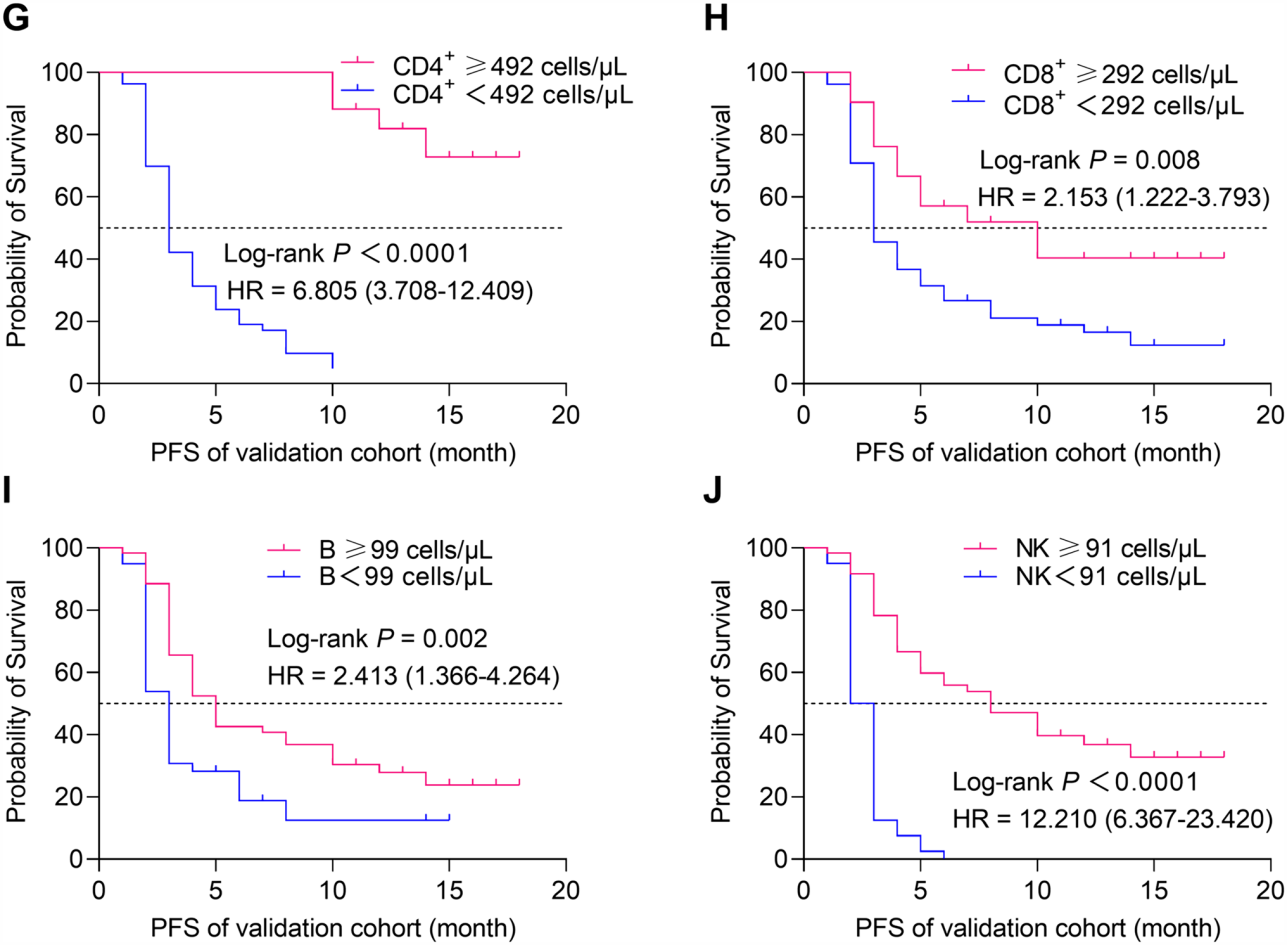
The relationship between ACLS and PFS in the training and validation cohorts. *P*-values were calculated by the log-rank test. The Kaplan–Meier analysis of the absolute counts of (**A**) CD3^+^, (**B**) CD4^+^, (**C**) CD8^+^, (**D**) B, and (**E**) NK cells in training cohort (n = 200). The Kaplan–Meier analysis of the absolute counts of (**F**) CD3^+^, (**G**) CD4^+^, (**H**) CD8^+^ AC, (**I**) B, and (**J**) NK cells in validation cohort (n = 100)

To further investigate the correlation between ACLS and PFS in patients with advanced NSCLC, firstly, X-tile software was used to obtain the optimal cutoff values of AC of CD3^+^ (898 cells/μL), CD4^+^ (492 cells/μL), CD8^+^ (292 cells/μL), B (99 cells/μL) and NK cells (91 cells/μL); then the patients were divided into high lymphocyte subsets and low lymphocyte subsets groups according to the cutoff value, and the differences in PFS between the two groups were compared. Kaplan–Meier survival curves showed that the higher AC of CD3^+^ (training cohort: HR 6.624, 95% CI 4.528–9.690,* P* < 0.0001; validation cohort: HR 5.518, 95% CI 3.064–9.939,* P* < 0.0001), CD4^+^ (training cohort: HR 8.443, 95% CI 5.777–12.310,* P* < 0.0001; validation cohort: HR 6.805, 95% CI 3.708–12.409,* P* < 0.0001), CD8^+^ (training cohort: HR 2.443, 95% CI 1.702–3.479,* P* < 0.0001; validation cohort: HR 2.153, 95% CI 1.222–3.793,* P* = 0.008), B (training cohort: HR 4.528, 95% CI 2.769–5.819, *P* < 0.0001; validation cohort: HR 2.413, 95% CI 1.366–4.264, *P* = 0.002) and NK cells (training cohort: HR 2.974, 95% CI 1.932–4.580,* P* < 0.0001; validation cohort: HR 12.210, 95% CI 6.367–23.420,* P* < 0.0001) were significantly associated with longer PFS in training and validation cohorts (Fig. 3). Taken together, the results indicated that the higher the ACLS, the longer the PFS of the advanced NSCLC patients, vice versa.

### Univariate and multivariate analysis of efficacy of anti-PD-1 inhibitors

To further clarify the factors affecting the therapeutic response, we evaluated the relationship between clinicopathological characteristics and ACLS and curative effect of patients in the training cohort.

**Fig. 4:**
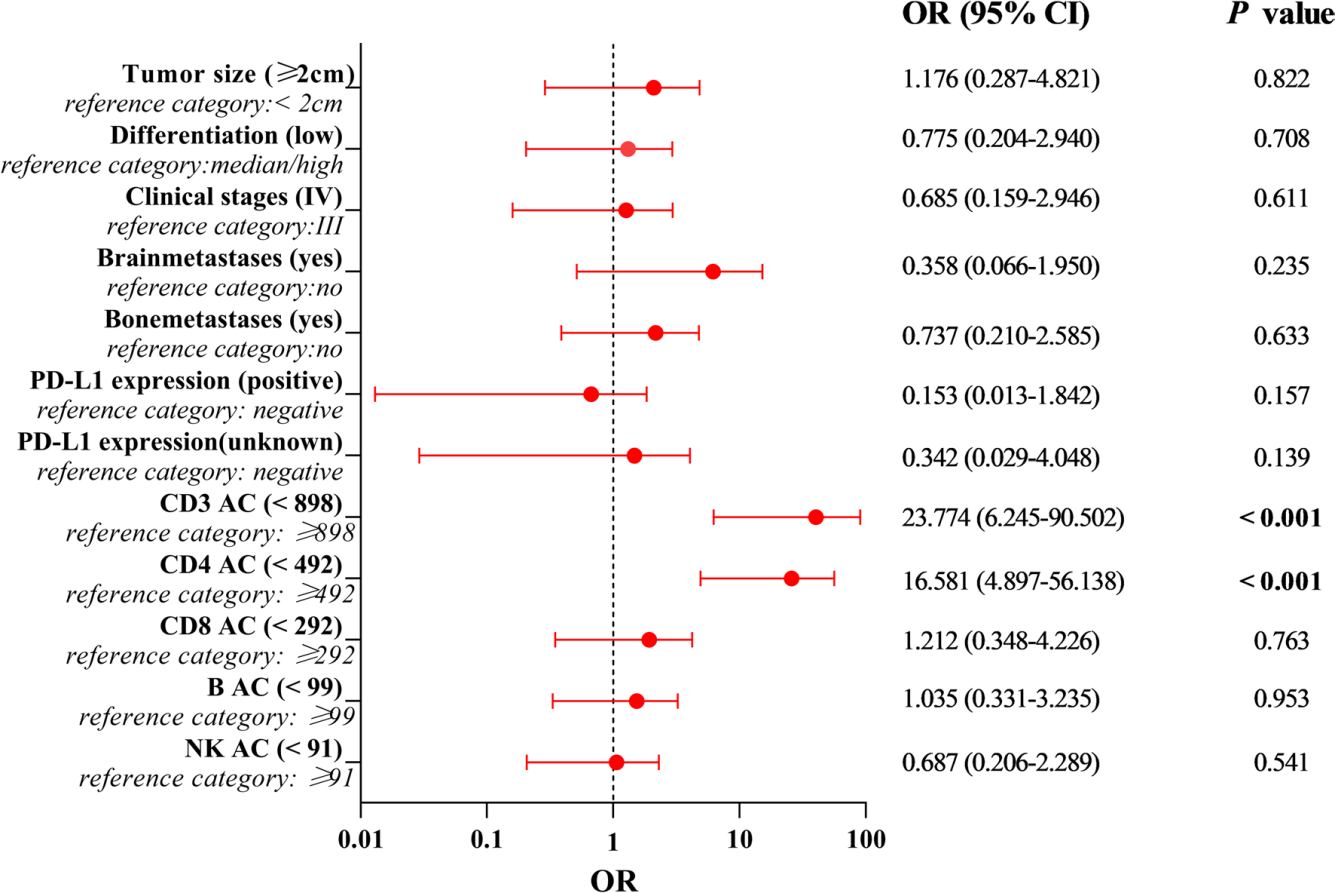
Forest plot of factors influencing the efficacy of aNSCLC patients. HR > 1 indicates that the variable is considered as a risk factor. HR < 1 represents that the variable is considered as a protective factor

Table 2 presents the results of logistic regression analysis in the training cohort. The univariate analysis revealed that tumor size ≥ 2 cm (*P* < 0.001), low differentiation (*P* = 0.01), clinical stages at IV (*P* < 0.001), brain metastases (*P* < 0.001), bone metastases (*P* = 0.028), PD-L1 expression < 1% (*P* < 0.001), and lower baseline AC of CD3^+^ (*P* < 0.001), CD4^+^ (*P* < 0.001), CD8^+^ (*P* < 0.001), B AC (*P* < 0.001) and NK cells (*P* = 0.002) were associated with poorer efficacy. The multivariate analysis showed that lower baseline AC of CD3^+^ (HR 23.774; 95% CI 6.245–90.502; *P* < 0.001) and CD4^+^ (HR 16.581; 95% CI 4.897–56.138; *P* < 0.001) were independent risk factors for poorer efficacy (Table 2 and Fig. 4).

**Table 2:** Univariate and multivariate logistic regression analysis of curative effect in the training cohort

Characteristics	Curative effect		Univariate analysis		Multivariate analysis	
	PR	SD + PD	χ^2^/Fisher	*P* value	OR (95% CI)	*P* value
Age(years)						
< 67	32	75	0.223	0.636		
≥ 67	25	68				
Gender						
Male	40	103	0.069	0.793		
Female	17	40				
Smoking history						
No	18	37	0.665	0.415		
Yes	39	106				
Tumor size						
< 2cm	17	12	15.1011	** < 0.001**	1 [Reference]	
≥ 2cm	40	131			1.176(0.287–4.821)	0.822
Histology						
Adenocarcinoma	32	91	0.976	0.614		
Squamous carcinoma	19	40				
Others	6	12				
Differentiation						
Low	39	121	6.680	**0.01**	1 [Reference]	
Median/high	18	22			0.775(0.204–2.940)	0.708
Clinical stages						
III	25	18	23.615	** < 0.001**	1 [Reference]	
IV	32	125			0.685(0.159–2.946)	0.611
Lymphatic metastasis						
No	13	32	0.004	0.948		
Yes	44	111				
Brain metastases						
No	54	96	16.562	** < 0.001**	1 [Reference]	
Yes	3	47			0.358(0.066–1.950)	0.235
Bone metastases						
No	40	76	4.851	**0.028**	1 [Reference]	
Yes	17	67			0.737(0.210–2.585)	0.633
Hepatic metastases						
No	47	100	3.283	0.070		
Yes	10	43				
PD-L1 expression						
Negative	15	87	19.438	** < 0.001**	1 [Reference]	
Positive	39	52			0.153 (0.013–1.842)	0.157
Unknown	3	4			0.342(0.029–4.048)	0.139
CD3^+^ AC						
≥ 898	40	5	103.914	** < 0.001**	1 [Reference]	
< 898	17	138			23.774(6.245–90.502)	** < 0.001**
CD4^+^ AC						
≥ 492	45	12	99.562	** < 0.001**	1 [Reference]	
< 492	12	131			16.581(4.897–56.138)	** < 0.001**
CD8^+^ AC						
≥ 292	29	34	13.872	** < 0.001**	1 [Reference]	
< 292	28	109			1.212(0.348–4.226)	0.763
B AC						
≥ 99	44	51	28.185	** < 0.001**	1 [Reference]	0.953
< 99	13	92			1.035(0.331–3.235)	
NK AC						
≥ 91	49	91	9.676	**0.002**	1 [Reference]	
< 91	8	52			0.687(0.206–2.289)	0.541

In univariate and multivariate binary logistic regression analysis, the bolded values represent that the *P* value was significant

### Univariate and multivariate Cox regression analyses of PFS

**Fig. 5:**
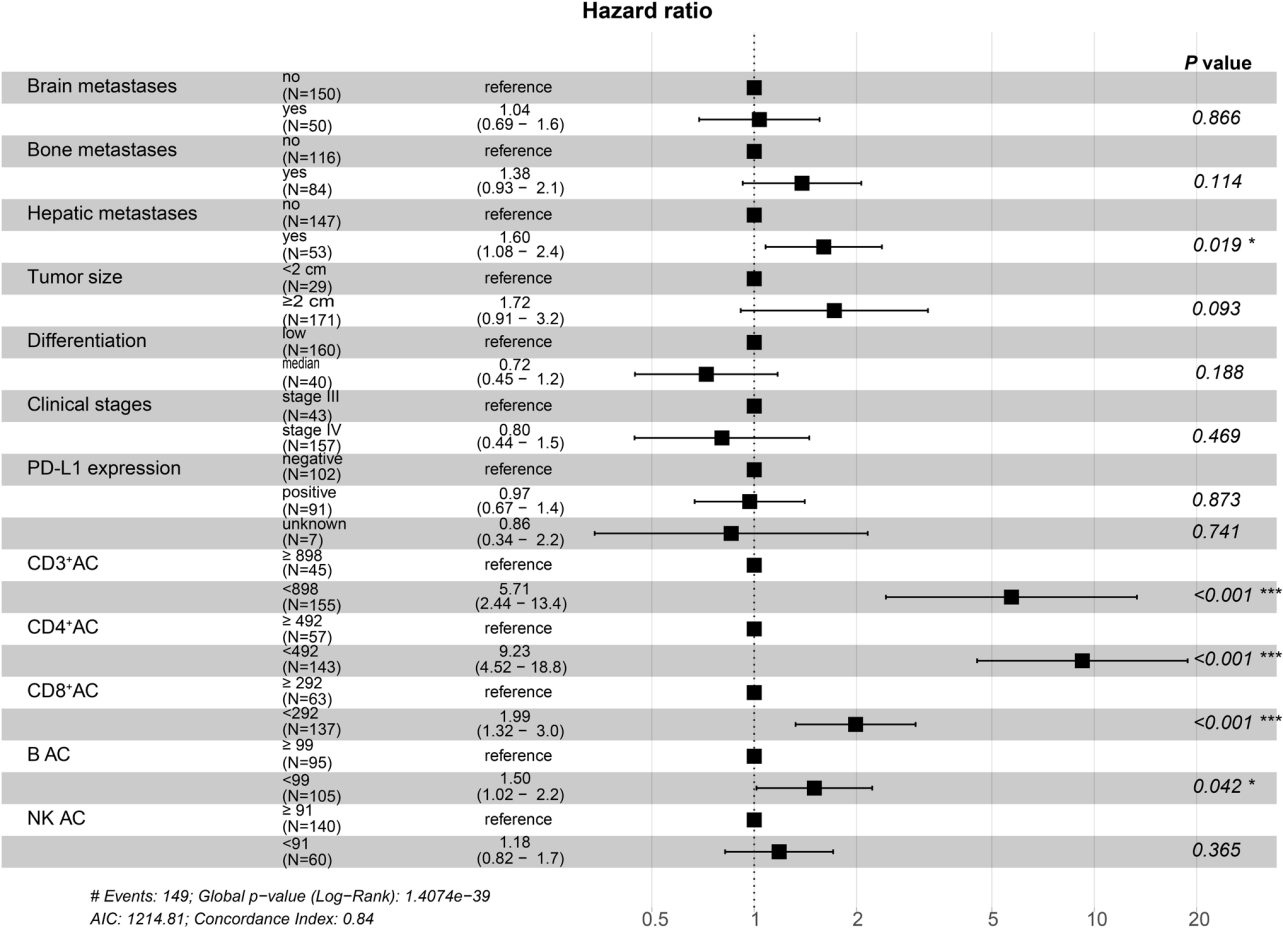
Forest plot of factors influencing PFS of aNSCLC patients. *P < 0.05; **P < 0.01; ***P < 0.001

To determine the prognostic factors associated with PFS, univariate and multivariate Cox proportional hazard regression analyses were performed. In univariate analysis, tumor size ≥ 2 cm (*P* = 0.001), low differentiation (*P* = 0.005), NSCLC patients with stage IV (*P* < 0.001), brain metastases (*P* < 0.001), bone metastases (*P* = 0.017), hepatic metastases (*P* < 0.001), PD-L1 expression < 1% (*P* < 0.001), and lower baseline AC of CD3^+^ (*P* < 0.001), CD4^+^(*P* < 0.001), CD8^+^ (*P* < 0.001), B (*P* < 0.001) and NK cells (*P* < 0.001) were associated with shorter PFS. (Table 3). Multivariate analysis showed that hepatic metastases (HR 1.602; 95% CI 1.082–2.421; *P* = 0.019) and lower baseline AC of CD3^+^ (HR 5.714; 95% CI 2.440–13.414; *P* < 0.001), CD4^+^ (HR 9.229; 95% CI 4.518–18.822; *P* < 0.001), CD8^+^ (HR 1.992; 95% CI 1.321–2.994; *P* < 0.001), B (HR 1.501; 95% CI 1.021–2.211; *P* = 0.042) were independent prognostic factors of shorter PFS (Table 3, Fig. 5).

**Table 3:** Univariate and multivariate Cox regression analysis of PFS

Characteristics	Univariate analysis		Multivariate analysis	
	HR (95% CI)	*P* value	HR (95% CI)	*P* value
Age(years)				
< 67	1 [Reference]			
≥ 67	1.107 (0.803–1.527)	0.535		
Gender				
Male	1 [Reference]			
Female	0.917 (0.643–1.307)	0.631		
Smoking history				
No	1 [Reference]			
Yes	1.240 (0.860–1.787)	0.249		
Tumor size				
< 2 cm	1 [Reference]			
≥ 2 cm	2.678 (1.536–4.667)	**0.001**	1.722 (0.909–3.211)	0.093
Histology				
Adenocarcinoma	1 [Reference]			
Squamous carcinoma	0.894 (0.617–1.295)	0.553		
Others	1.098 (0.637–1.895)	0.746		
Differentiation				
Low	1 [Reference]			
Median/high	0.536 (0.345–0.831)	**0.005**	0.719 (0.452–1.224)	0.118
Clinical stages				
III	1 [Reference]			
IV	2.948 (1.828–4.755)	** < 0.001**	0.813 (0.443–1.528)	0.469
Lymphatic metastasis				
No	1 [Reference]			
Yes	1.090 (0.733–1.620)	0.669		
Brain metastases				
No	1 [Reference]			
Yes	2.105 (1.474–3.006)	** < 0.001**	1.040(0.690–1.600)	0.866
Bone metastases				
No	1 [Reference]			
Yes	1.485 (1.074–2.053)	**0.017**	1.380(0.930–2.100)	0.114
Hepatic metastases				
No	1 [Reference]			
Yes	1.873 (1.320–2.659)	** < 0.001**	1.602(1.082–2.421)	**0.019**
PD-L1 expression				
Negative	1 [Reference]			
Positive	0.522 (0.373–0.732)	** < 0.001**	0.912 (0.671–1.416)	0.873
Unknown	0.630 (0.255–1.556)	**0.317**	0.860 (0.344–2.211)	0.741
CD3^+^ AC				
≥ 898	1 [Reference]			
< 898	16.934(7.717–37.160)	** < 0.001**	5.714(2.440–13.414)	** < 0.001**
CD4^+^ AC				
≥ 492	1 [Reference]			
< 492	15.417(8.141–29.195)	** < 0.001**	9.229(4.518–18.822)	** < 0.001**
CD8^+^ AC				
≥ 292	1 [Reference]			
< 292	2.339 (1.599–3.421)	** < 0.001**	1.992(1.321–2.994)	** < 0.001**
B AC				
≥ 99	1 [Reference]			
< 99	3.170 (2.232–4.503)	** < 0.001**	1.501(1.021–2.211)	**0.042**
NK AC				
≥ 91	1 [Reference]			
< 91	2.619 (1.540–3.055)	** < 0.001**	1.182(0.822–1.730)	0.365

In univariate and multivariate Cox analysis, the bolded values represent that the *P* value was significant

### Construction of nomograms for predicting efficacy and PFS

**Fig. 6:**
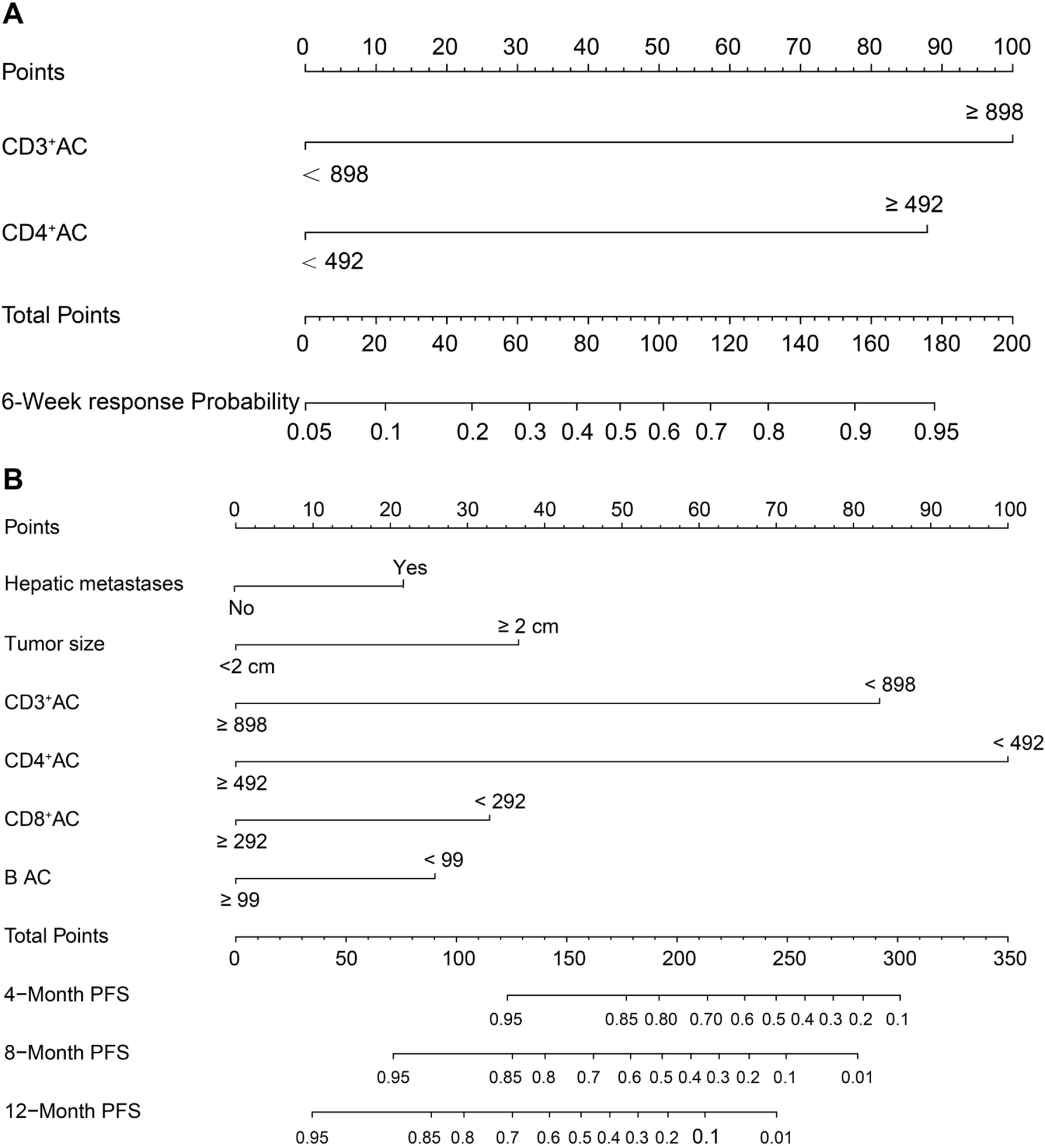
The nomograms for predicting efficacy and PFS in advanced NSCLC patients. **A** Nomogram was developed based on two factors including baseline AC of CD3^+^ and CD4^+^ to predict probability of therapeutic response at 6-weeks after treatment. **B** Nomogram was developed based on six factors including hepatic metastases, tumor size, and baseline AC of CD3^+^, CD4^+^, CD8^+^ to predict probability of PFS at 4-, 8- and 12-months

Based on the independent risk factors obtained from the training cohort, we established two nomograms to predict therapeutic response and PFS respectively. The first nomogram was constructed to predict probability of therapeutic response at 6-weeks after treatment based on baseline AC of CD3^+^ and CD4^+^ (Fig. 6A). The second nomogram was built to predict probability of PFS at 4-, 8- and 12-months for aNSCLC patients treated with anti-PD-1 antibodies based on hepatic metastases, tumor size, and baseline AC of CD3 + , CD4 + , CD8^+^ and B cells (Fig. 6B).

From the nomograms, we can see that the independent factors can be assigned a corresponding score for each patient. By summing the scores of the variables and locating them on the total points, we can easily draw a straight line down to determine the probability of response at 6-weeks after anti-PD-1 inhibitors treatment and probability of PFS at 4-, 8- and 12-months for each advanced NSCLC patient.

### Calibration and validation of the nomograms

**Fig. 7:**
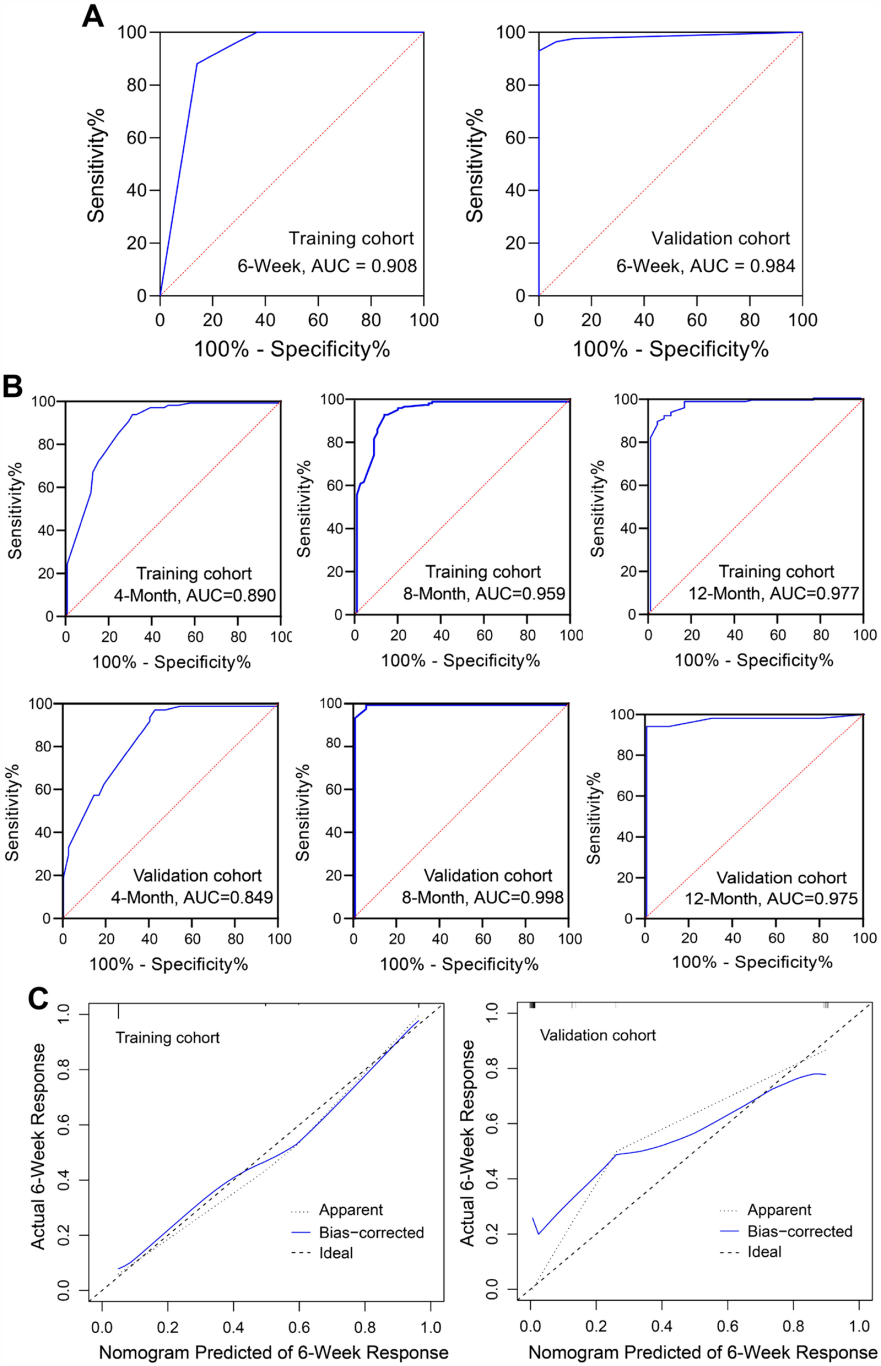

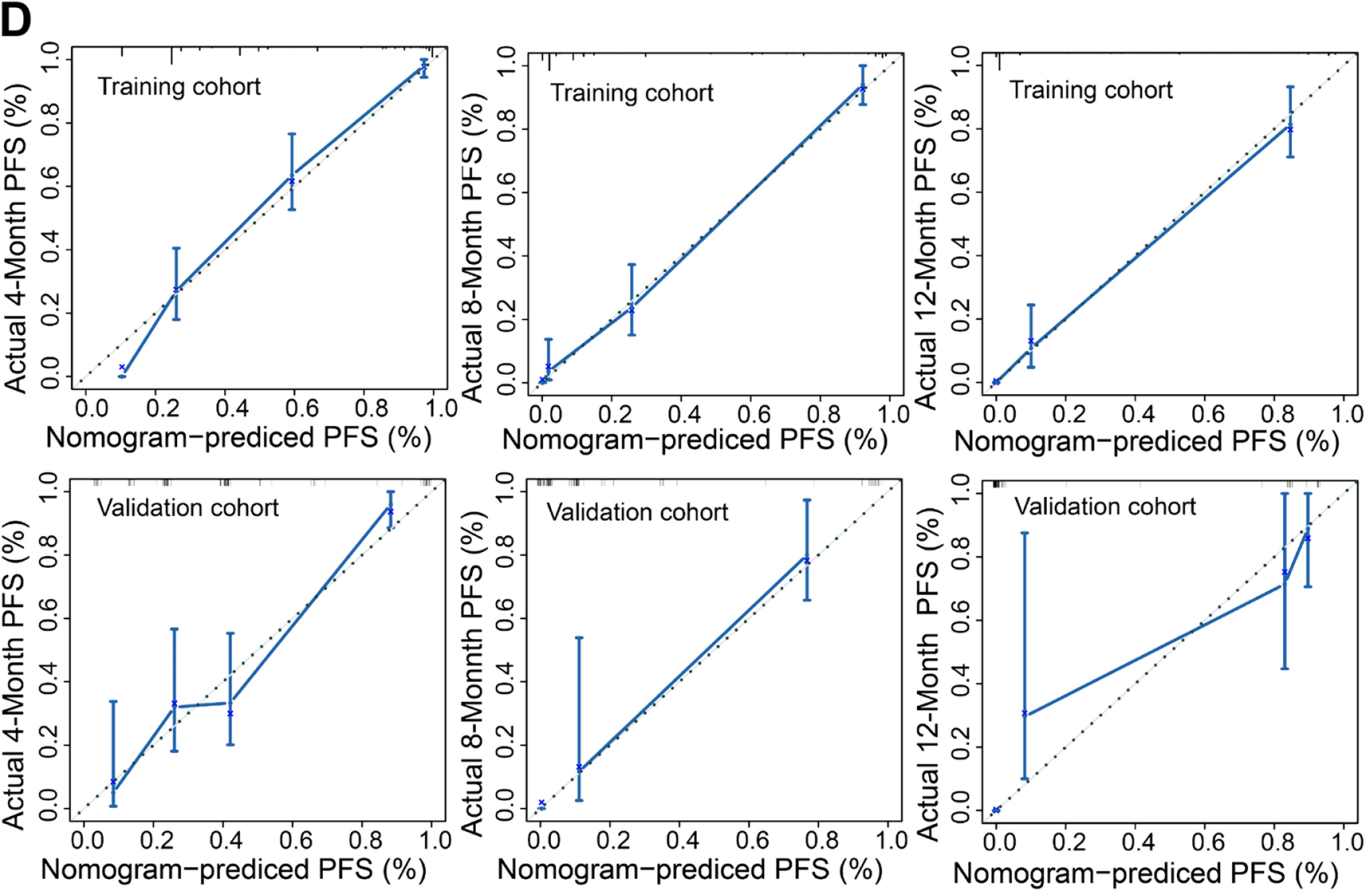
ROC curves and calibration curves of the nomograms for predicting efficacy and PFS in the training and validation cohorts. **A** ROC curves of the nomograms to predict therapeutic response at 6-weeks in both training and validation cohort. **B** ROC curves of the nomogram for predicting 4-, 8-, and 12-months PFS in the training and validation cohorts. **C** Calibration curves of nomogram for predicting therapeutic response at 6-weeks in training and validation cohorts. **D** Calibration curves of nomogram for predicting 4-, 8-, and 12-months PFS in training and validation cohorts

To assess the accuracy and discrimination of the nomograms, the concordance index (C-index), calibration curves, and the ROC analysis were performed. The area under the ROC curve (AUC-ROC) of nomogram to predict response was 0.908 (95% CI 0.851–0.965) in training cohort and 0.984 (95% CI 0.963–1.00) in validation cohort (Fig. 7A). The C-index of nomogram to predict PFS was 0.825(95% CI 0.755–0.896) in training cohort and 0.832(95% CI 0.762–0.901) in validation cohort. The AUC-ROC showed an excellent discriminative ability in both cohorts (training cohort: 4-months AUC-ROC 0.890, 95% CI 95% CI 0.847–0.932; 8-months AUC-ROC 0.959, 95% CI 0.932–0.986; 12-months AUC-ROC 0.977, 95% CI 0.957–0.998; validation cohort: 4-months AUC-ROC 0.849, 95% CI 0.775–0.923; 8-months AUC-ROC 0.998, 95% CI 0.993–1.00; 12-months AUC-ROC 0.975, 95% CI 0.99–1.00) (Fig. 7B). Moreover, the calibration curves of each nomogram demonstrated a superior consistence between the nomogram predicted probability and actual observation for predicting 6-weeks therapeutic response and 4-, 8-, and 12-months PFS both in the training and validation cohorts (Fig. 7C, D).

## Discussion

In recent years, the advent of ICI is rapidly changing the treatment prospective for NSCLC patients. However, the efficacy of anti-PD-1 inhibitors treatment varies widely among NSCLC patients. Therefore, the screening of potential patients who would benefit from anti-PD-1 inhibitors has become a new challenge. To accurately predict the prognosis and the efficacy of anti-PD-1 inhibitors, we investigated the predictors influencing the efficacy and prognosis and developed two nomograms based on ACLS to predict the efficacy after 6 weeks of treatment with anti-PD-1 inhibitors and the probability of PFS at 4-, 8- and 12 months, respectively.

Herein, we identified 6 factors, including hepatic metastases, tumor size, AC of CD3^+^, CD4^+^, CD8^+^, and B cells, were correlated with the efficacy of anti-PD-1 inhibitors and PFS in training cohort. Our study revealed that the two nomograms based on ACLS all have a good ability of concordance and discrimination, and their performance has been rigorously validated internally and externally. Compared to other single biomarkers or risk scores, our nomograms have the following advantages. Firstly, the nomograms can quantify the predictors and provide individualized predictive probabilities of treatment response and prognosis for each patient. Secondly, the nomograms incorporated ACLS into the construction of the model for the first time, and the variables required for model building are easier to obtain. Finally, the nomograms have superior sensitivity and specificity, and are constructed based on real-world clinical data, which have unique clinical application and promotion advantages.

Peripheral blood lymphocyte subsets can directly reflect the immune status of the body. Hence, it is vital to include baseline ACLS in the development of the nomograms for aNSCLC patients. Firstly, functionally, lymphocyte subsets have the functions of cellular immunity and humoral immunity, such as CD3^+^ , CD4^+^ , CD8^+^ and NK cells play an important role in cellular immunity, while B cells have a key role in humoral immunity. Secondly, developmentally, they encompass both intrinsic and adaptive immunity, such as NK cells belong to intrinsic immunity, while CD3^+^, CD4^+^, CD8^+^ and B cells belong to adaptive immunity. Thirdly, in terms of cellular interactions, lymphocyte subsets include antigen-presenting cells (B cells), CD4^+^ helper cells and killer cells (CD8^+^ and NK cells), CD4^+^ helper cells have an important regulatory effect on other immune cells [30]. Fourthly, the CD4^+^/CD8^+^ ratio is an indicator of immune homeostasis. Fifthly, the percentage of lymphocyte subsets is the ratio and composition of each subsets, which reflects the development and differentiation of lymphocytes. The ACLS are the numbers of each subsets, which reflect the proliferation ability of lymphocytes. Sufficient numbers of immune cells are a prerequisite for killing tumor cells [26].

Absolute lymphocyte counts can be used as an indicator of host immune status and were closely related to the efficacy of immune checkpoint inhibitors and OS of melanoma patients [31]. Another study shown that lymphopenia is an independent prognostic factor for OS and PFS in advanced breast carcinoma, sarcomas, and non-Hodgkin's lymphomas [32]. The study further investigated the lymphocyte subsets and suggested that patients with CD3^+^AC < 898 cells/μL, CD4^+^AC < 498 cells/μL, CD8^+^AC < 292 cells/μL, B AC < 99 cells/μL and NK AC < 91 cells/μL had significantly poorer efficacy and PFS than those with CD3^+^AC ≥ 898 cells/μL, CD4^+^AC ≥ 498 cells/μL, CD8^+^AC ≥ 292 cells/μL, B AC ≥ 99 cells/μL and NK AC ≥ 91 cells/μL, indicating that the baseline peripheral blood ACLS significantly affected the efficacy of anti-PD-1 inhibitors and prognosis of aNSCLC patients. It is worth noting that the CD3^+^AC < 898 cells/μL and CD4^+^AC < 498 cells/μL were independent risk factors for the efficacy. Hepatic metastasis, tumor size ≥ 2 cm, CD3^+^AC < 898 cells/μL, CD4^+^AC < 498 cells/μL, CD8^+^AC ≥ 292 cells/μL and B AC ≥ 99 cells/μL were independent risk factors affecting PFS.

The common metastatic sites of NSCLC include brain, bone, liver, and adrenal glands, and about 20% of NSCLC patients have liver metastases [33]. Study showed that NSCLC patients without liver metastases treated with nivolumab had a significantly longer median PFS than those with liver metastases (100 days vs. 42 days, log-rank *P* = 0.0002) [34]. Another study revealed that advanced lung cancer patients with liver metastases treated with durvalumab had shorter OS and PFS than those without liver metastases, and with a significantly lower ORR [35]. Daud et al. suggested that liver metastasis was associated with reduced response and shortened progression-free survival (PFS; ORR 30.6%; median PFS 5.1 months) compared with NSCLC patients without liver metastasis (ORR 56.3%; median PFS 20.1 months) *P* ≤ 0.0001 [36]. In present study, multivariate Cox regression analysis showed that liver metastasis was an independent risk factor for PFS, and the risk of progression in aNSCLC patients with liver metastasis was 1.873 times (95% CI 1.320–2.659; *P* = 0.019) higher than that in patients without liver metastasis. Consequently, it was crucial to include liver metastases in the construction of nomograms to predict PFS.

Tumor size was an important factor affecting the response to radiotherapy and immunotherapy in NSCLC patients and was identified as an independent prognostic factor of worse OS and PFS [37, 38]. Study demonstrated that NSCLC patients with large baseline tumors treated with immune checkpoint inhibitor had remarkably shorter PFS than patients with small baseline tumors (median PFS 2.07 months [95% CI 0.99–6.77] versus 6.39 months [95% CI 4.17–11.50], *P* = 0.044), and NSCLC patients with large baseline tumors also had significantly shortened OS (*P* < 0.0001) [39]. Our study observed similar results that the risk of progression in advanced NSCLC patients with tumors ≥ 2 cm was 2.678 times (95% CI 1.536–4.667) higher than that in patients with tumors < 2 cm.

There were some limitations in the present study. Firstly, the study was a single-center retrospective study with a relatively small sample size, so some information bias may exist, and a multicenter prospective study with a large sample is needed to further validate our results. Secondly, we could not include all potential influencing factors in our analysis due to the limited sample size.

## Conclusions

The study shed light on ACLS were closely correlated with the efficacy and PFS of aNSCLC patients treated by anti-PD-1 inhibitors, the higher ACLS, the better efficacy and longer PFS, vice versa. Meanwhile, we determined 2 predictors affecting efficacy and 6 predictors affecting PFS from clinicopathological characteristics and immunological indicators. Based on these factors, we constructed and validated two different nomograms to predict the probability of efficacy at 6 weeks after treatment and the probability of PFS at 4-, 8- and 12- months in aNSCLC patients treated with anti-PD-1 inhibitors, respectively. The two models are important guides for prediction of prognosis and individualized diagnosis and treatment decisions, and have a potent clinical value for application.

## Data Availability

The datasets generated during and/or analysed during the current study are available from the corresponding author on reasonable request.

## Abbreviations

AC: Absolute count
ACLS: Absolute counts of lymphocyte subsets
DDR: DNA damage repair
MSI: Microsatellite instability
NSCLC: Non-small-cell lung cancer
ORR: Objective response rate
OS: Overall survival
PFS: Progression-free survival
PD-L1: Programmed death protein ligand-1
PFS: Progression-free survival
TMB: Tumor mutation burden
TILs: Tumor-infiltrating lymphocytes
